# Patent urachus presenting as persistent umbilical discharge with concomitant umbilical hernia in an adult: a case report

**DOI:** 10.1093/bjrcr/uaag047

**Published:** 2026-11-05

**Authors:** Aistė Pileckienė, Ingrida Pikūnienė, Vaida Atstupėnaitė, Gintaras Pikūnas, Eglė Stanišauskytė

**Affiliations:** Department of Radiology, Lithuanian University of Health Sciences, Kaunas, LT-50161, Lithuania; Department of Radiology, Lithuanian University of Health Sciences, Kaunas, LT-50161, Lithuania; Department of Radiology, Lithuanian University of Health Sciences, Kaunas, LT-50161, Lithuania; Department of Surgery, Public Institution Raseiniai Hospital, Raseiniai, LT-60127, Lithuania; Department of Radiology, Lithuanian University of Health Sciences, Kaunas, LT-50161, Lithuania

**Keywords:** patent urachus, urachal anomalies, umbilical discharge, case report

## Abstract

Patent urachus (PU) is usually diagnosed in the neonatal or early pediatric period, while adult cases are uncommon. We report a 26-year-old woman with intermittent umbilical urine leakage, mild periumbilical erythema, tenderness and serous discharge. MRI revealed a tubular tract connecting the bladder dome to the umbilicus, suggestive of PU. The patient underwent laparoscopic excision of the urachal remnant with primary repair of a small umbilical hernia. Histopathological examination confirmed the diagnosis. This case demonstrates that PU may persist into adulthood and present with nonspecific symptoms, requiring appropriate imaging for accurate diagnosis and definitive surgical treatment.

## Introduction

The urachus is a tubular-shaped structure located between the umbilicus and the urinary bladder.^1^ During the first trimester of fetal development, the urachus is vital in draining urine from the fetus’s bladder.^2^ This tube begins to obliterate around the fourth and fifth months of gestation, usually finishing by birth, and the median umbilical ligament forms in its place, connecting the apex of the urinary bladder to the umbilicus.^1^ When this crucial closing process fails to occur properly, it results in a group of rare congenital disorders known as urachal anomalies (UA).^3^ Urachal anomalies are classified into 4 types: urachal cyst, patent urachus (PU), urachal sinus, and urachal diverticulum.^4^ Among these, PU is considered one of the rarest subtypes, although its reported frequency varies across studies. Recent studies with larger patient cohorts suggest that PU remains an uncommon presentation.^2^^,^^5–7^ This condition specifically involves the complete failure of the fetal channel between the urinary bladder and the umbilicus to close during gestation, leaving an open communication (fistula) between the urinary bladder and the umbilicus.

With the increasing use of imaging modalities, UAs are now recognized as more common than previously thought. Although the overall prevalence of UA in the pediatric population undergoing radiological evaluation is 1.03%, PU constitutes only 1.5% of these cases.^5^

The persistence of an embryonic urachal remnant can give rise to various clinical problems, not only in infants and children but also in adults. Clinically, PU usually presents with urine leakage from the umbilicus, which may cause local skin irritation, dermatitis, cellulitis and increase the risk of urinary tract infections, abscess formation and sepsis due to the persistent communication between the bladder and the external environment.^2^ To prevent recurrent infections and potential malignant transformation, surgical or laparoscopic excision of PU is required.^6^

## Case presentation

A 26-year-old female with no significant past medical history presented with about a 2-month history of chronic, intermittent, serous discharge from the umbilicus, associated with periumbilical erythema, mild edema, and localized tenderness. The patient had attempted self-administered conservative management at home, maintaining regular umbilical hygiene with topical hydrogen peroxide. The patient was afebrile, and laboratory workup, including complete blood count (CBC) and C-reactive protein (CRP) and urinalysis was within normal limits. While the abdomen was soft, non-distended, and non-tender, inspection of the umbilicus revealed a small umbilical hernia, with minimal erythema and serous discharge. Because of the suspected umbilical hernia, the patient was admitted for a comprehensive diagnostic evaluation.

## Imaging

An abdominal ultrasound (US) was performed; however, no definite umbilical hernia was visualized. The examination revealed an oval hypoechoic structure measuring 4 × 8 mm anterior to the urinary bladder wall, but did not clearly demonstrate a definite communication between the bladder dome and the umbilicus (Figure 1). Therefore, MRI was performed to allow more precise anatomical evaluation. There were no signs of infection, and findings of physical examination were suggestive of an umbilical hernia, so the MRI was performed without administration of an intravenous gadolinium binding contrast agent. T2-weighted (T2W) sequences acquired in axial, coronal, and sagittal planes were used. MRI demonstrated a localized 4 × 13 mm collection of high-signal-intensity in T2W viscous fluid adjacent to the umbilicus, along with a slender, tubular, cord-like structure extending from the apex of the urinary bladder to the umbilicus. These findings were highly suggestive of a PU (Figures 2 and 3).

**Figure 1:**
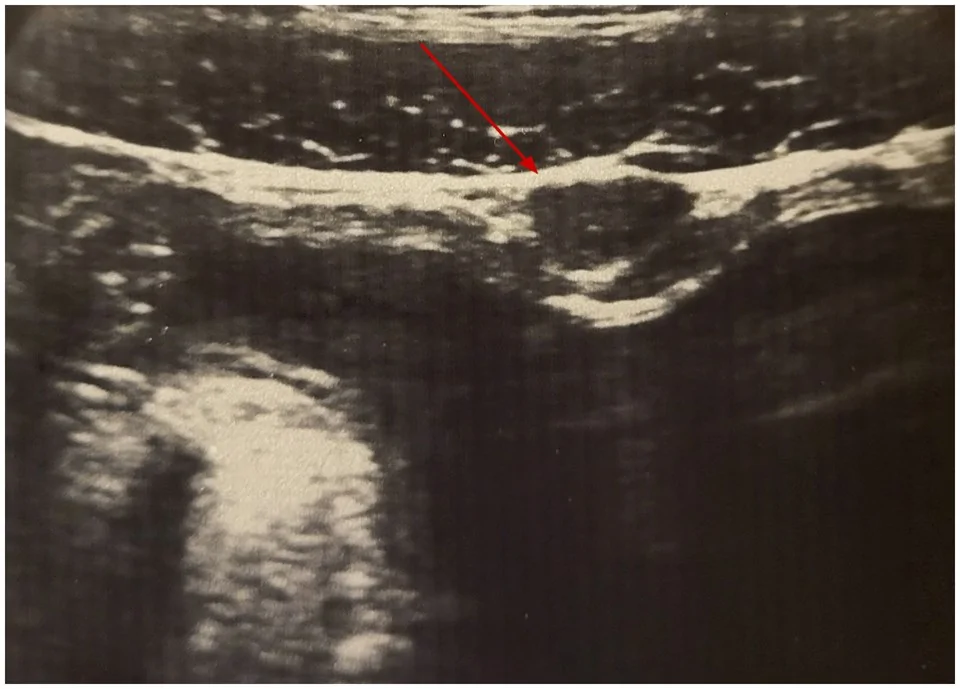
Ultrasound (US) demonstrates an oval hypoechoic structure (arrow) in front of the fundus of the urinary bladder.

**Figure 2:**
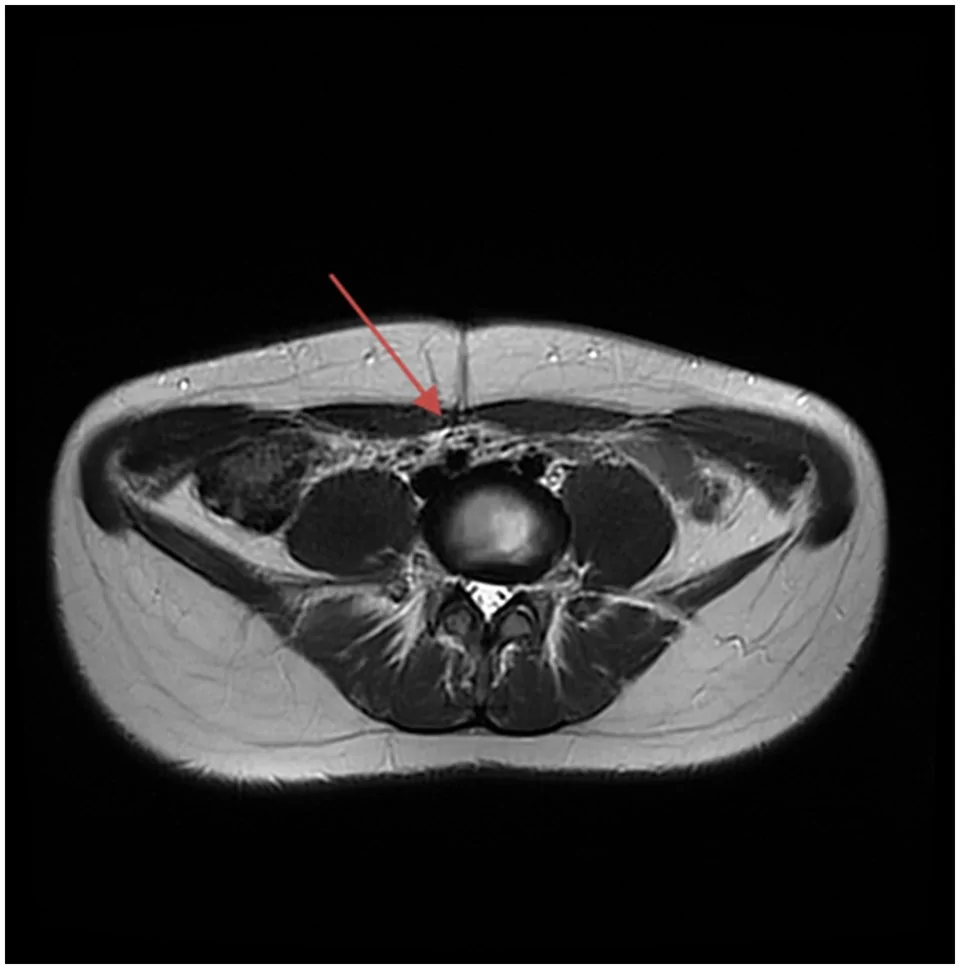
Sagittal T2-weighted MRI demonstrates a tubular structure (arrow) connecting the dome of the urinary bladder to the anterior abdominal wall.

**Figure 3:**
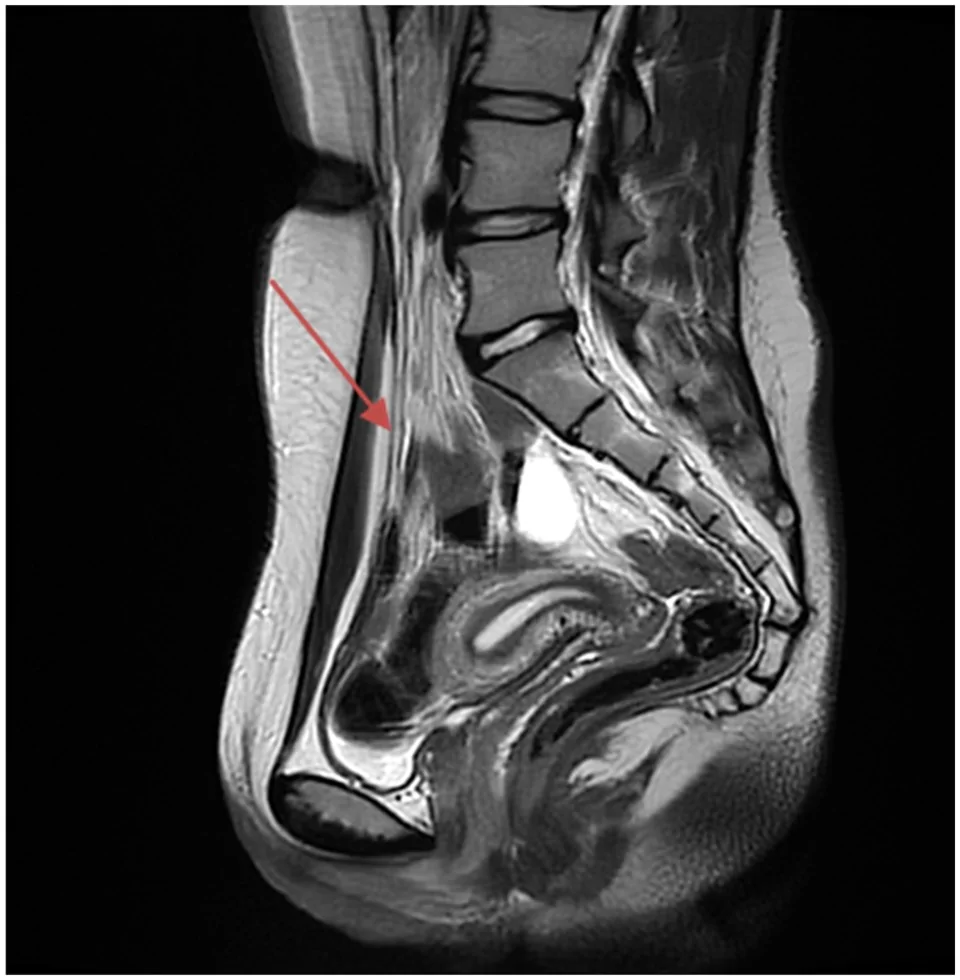
Axial T2-weighted MRI demonstrates a round tubular structure (arrow) extending to the anterior abdominal wall.

## Management and final diagnosis

The operation was performed under general anesthesia. The open approach was chosen for repair of the umbilical hernia, while the PU was excised laparoscopically, primarily for cosmetic reasons. Laparoscopically, the urachal tract was identified extending from the umbilicus toward the urinary bladder, dissected from the surrounding tissues, clipped near the bladder dome, and excised (Figure 4). Following instillation of 300 mL of methylene blue solution through a Foley catheter, no leakage was observed.

**Figure 4:**
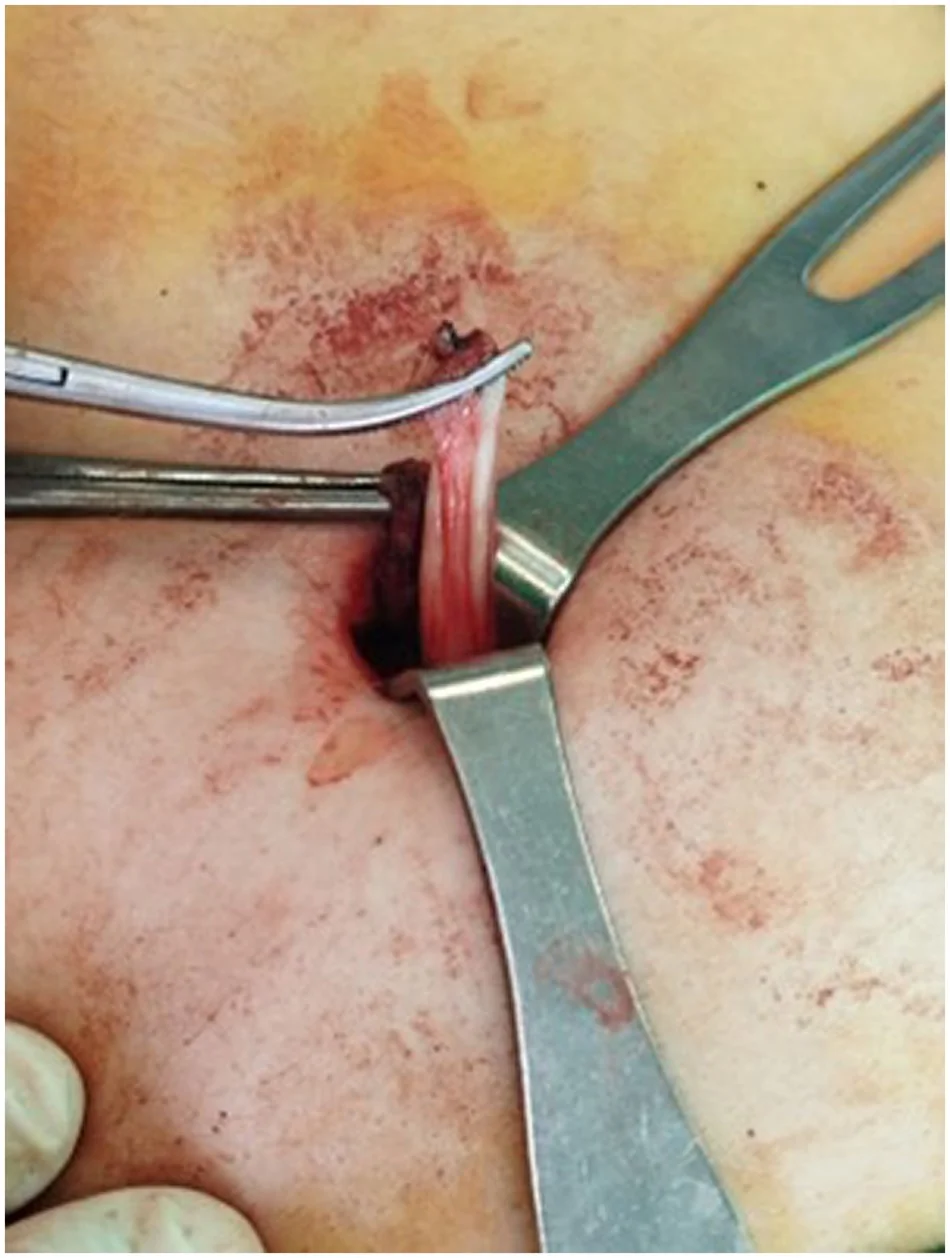
Urachus excision surgery.

The concomitant umbilical hernia was repaired using an open approach. A 1.5-cm hernia defect with residual urachal and granulation tissue was identified. The residual tissue was excised, the hernia sac was reduced, and primary tissue repair was performed without mesh. An inverted hernioplasty was performed using the patient’s own tissue. The procedure was completed without complications.

Postoperatively, the patient received analgesics, antiemetics, and prophylactic antibiotics. Histopathological examination confirmed a benign PU composed of smooth muscle, fibrous and adipose tissue containing duct-like structures lined by transitional epithelium. Correlation of clinical, radiological, and histological findings established the final diagnosis of PU. At the 3-month follow-up, excellent wound healing and complete resolution of symptoms were observed.

## Discussion

Patent urachus is an exceptionally rare urachal anomaly that may present at any age, with the majority of cases identified in the neonatal or early pediatric period due to visible umbilical urine discharge. However, as seen in this case, a subset of patients remains asymptomatic for decades, with clinical manifestation occurring later in young adulthood. In the present case, the patient presented with serous discharge from the umbilicus. The differential diagnosis of umbilical discharge includes several congenital and acquired conditions, with the nature of the discharge providing useful diagnostic clues. Purulent or foul-smelling discharge is typically associated with omphalitis, an umbilical abscess, or an infected urachal sinus. Clear or serous discharge may occur with a PU, particularly when it represents urine, while serous discharge may also be seen in association with an umbilical hernia. Bloody or cyclic discharge in women of reproductive age may raise suspicion of umbilical endometriosis.^8–10^ Because of its rarity, the true prevalence of PU in adults is difficult to ascertain; adult presentations are reported only sporadically and comprise a very small proportion of urachal pathology. Across neonatal/pediatric series, UAs are estimated at roughly 1 in 5000-8000 live births, and only a small fraction persist or present in adulthood.^11^ Adult-specific data remain limited, with hospital-based figures suggesting urachal disease in adults is uncommon (≈2 per 100 000 hospitalizations).^8^

Management of UAs is based on the potential for these remnants to become symptomatic or develop complications. Firstly, PU creates a direct pathway for bacterial distribution that can lead to various infections or intraperitoneal abscesses.^2^ Secondly, chronic inflammation and possible epithelial metaplasia of the urachal remnant may increase the risk of malignant transformation, often diagnosed only at advanced stages due to a lack of early, specific symptoms.^9^^,^^12^^,^^13^ For these reasons, complete surgical excision of the urachal tract, including a cuff of the bladder dome, is strongly recommended, even in asymptomatic cases or incidentally detected remnants.

Given these significant risks, diagnostics are crucial. Imaging modalities, such as US, MRI, or CT, are used to confirm the diagnosis. Radiographic imaging plays a main role in establishing the diagnosis, defining the extent of the tract, and excluding complications such as infection or neoplasia. In most cases, including ours, US is used as a primary imaging modality, as it is easily accessible, quick to perform, and cost-effective. The US can help to determine the location and extent of the UAs. For more in-depth visualization and to determine the possible complications, CT or MRI may be used.^7^ In the present case, the initial US examination did not clearly demonstrate a definite communication between the bladder dome and the umbilicus. Therefore, additional imaging modalities were required to determine the cause of the umbilical discharge and to allow more precise anatomical evaluation. MRI was preferred over CT due to its excellent soft-tissue resolution, multiplanar capability, and ability to distinguish fluid collections from surrounding tissues without exposure to ionizing radiation for a reproductive-age female.^14^ MRI also provides valuable information regarding the continuity between the bladder apex and the umbilicus, confirming the diagnosis of a PU. Although non-contrast MRI was sufficient to delineate the urachal tract in our patient, contrast-enhanced MRI may provide additional diagnostic information. It can help assess inflammatory changes and vascularity and may aid in the detection of infection, abscess formation, or neoplastic changes. It may also improve characterization of the urachal tract and surrounding soft tissues when non-contrast findings are inconclusive.^7^

In selected cases, fistulography may serve as a complementary diagnostic tool for PU. By injecting contrast material through the umbilical opening, fistulography can directly delineate the fistulous tract and confirm its communication with the urinary bladder.^14^ Another imaging approach is fluoroscopic cystography. It is performed by inserting a Foley catheter through the urethra into the urinary bladder, followed by gravity instillation of water-soluble iodinated contrast material until maximal bladder distention is achieved, allowing assessment of any communication between the urachal remnant and the bladder.^14^ Such investigations may provide additional confirmation of urachal patency and help clarify the anatomy when required for diagnostic or surgical planning. However, in the present case, fistulography and fluoroscopic cystography were not considered essential preoperatively because the diagnosis was established by MRI without the need for an additional invasive procedure.

The primary goal of surgical management for PU is definitive excision to eliminate the risk of complications and the possibility of malignant transformation. Although there are several approaches to the resection, the choice between laparoscopic and open techniques remains a subject of discussion.^15–17^ The surgical approach to urachal remnants has evolved over time and varies depending on patient age and institutional preference. In both pediatric and adult populations, laparoscopic excision has become increasingly adopted, offering advantages such as improved visualization of the operative field, easier access to the bladder dome, reduced postoperative morbidity, and favorable cosmetic outcomes.^15^^,^^17^^,^^18^ In the present case, a laparoscopic approach was selected based on these established benefits.

Only a limited number of PU cases have been documented in adults, and only a few published articles focus on its imaging findings, particularly MRI. Compared with the existing literature, this case demonstrates atypical features. Most reported UAs involve the pediatric population or adults who present only after developing complications such as infection or urachal cysts, abscesses, or urachal sinus tracts, rather than a fully patent channel.^10^ A similar case has been documented in the literature, where recent-onset umbilical discharge rapidly progressed to signs of systemic infection and colicky-like pain in the hypogastrium.^9^ In contrast, our patient had no evidence of infection, and imaging confirmed the presence of an isolated PU. This presentation aligns with another retrospective study where approximately 70% of patients with PU had umbilical discharge as a primary and sole symptom.^19^ In our case, the local erythema and pain were likely due to the chronic chemical irritation caused by the intermittent urine leakage.

Delayed diagnosis in adulthood has also been documented. Mirzaei et al. reported a male patient with a 20-year history of uninvestigated urinary leakage from the umbilicus. During evaluation for renal colic, PU was discovered incidentally, already containing a 6 mm calculus.^20^ This case emphasizes the need to consider UAs in adults with persistent or recurrent umbilical discharge, even in the absence of infection.

## Conclusion

Patent urachus represents one of the rarest types of UAs. This condition is usually diagnosed in the pediatric population, but can persist into adulthood with nonspecific symptoms, such as recurrent umbilical discharge, even without an infection. This case shows the diagnostic challenge in adults, where the US may not always be sufficient for accurate differential diagnosis; therefore, MRI can play a crucial role in accurate diagnosis and surgical planning. In our patient, complete surgical excision resulted in symptom resolution and an uncomplicated recovery.

## Learning points

- Patent urachus (PU), although typically diagnosed in childhood, should remain in the differential diagnosis of persistent or recurrent umbilical discharge in adults.
- Ultrasound may be insufficient to establish the diagnosis, whereas MRI allows more accurate assessment of the urachal tract and supports surgical planning.
- A laparoscopic approach offers safe and effective definitive treatment with excellent postoperative outcomes in adult patients with PU.
